# A Review of Community Awareness for Colorectal Cancer Screening and Prevention in North and Central Asian Countries

**DOI:** 10.7759/cureus.40540

**Published:** 2023-06-16

**Authors:** Maxwell Akanbi, Olga J Santiago Rivera, Arunima Dutta, Rebecca Pratiti

**Affiliations:** 1 Internal Medicine, McLaren Health Care, Flint, USA; 2 Graduate Medical Education, McLaren Oakland, Pontiac, USA; 3 Internal Medicine, Franciscan Health, Seattle, USA

**Keywords:** colon cancer prevention, health communication, screening, community awareness, colonic neoplasm

## Abstract

Objectives: Colorectal cancer (CRC) incidence and mortality rates are increasing in low- and middle-income countries (LMIC), including North and Central Asian countries (NCAC). Screening and risk factor reduction can aid in the prevention of colorectal cancer, but communities lack awareness of these screening programs. The review assessed community awareness about CRC screening and prevention in NCAC to facilitate cancer control policies.

Study type and methods: For this scoping review, we searched PubMed/Medline, Embase, and the Cochrane Library for articles on community awareness about CRC screening and prevention in NCAC according to inclusion and exclusion criteria.

Results: Eight of 677 articles from five of the 15 NCAC countries met the criteria. Most of the studies utilized a survey design. The results indicated low awareness of the availability of CRC screening and poor knowledge of CRC symptoms. Knowledge of CRC screening modalities was also inadequate. Some countries also lack CRC screening programs.

Conclusion: Community unawareness is a significant barrier to screening program utilization and sustenance. Community health awareness programs (CHAP) are needed to improve the uptake of CRC screening in NCAC. The NCAC should include CHAP as an integral component of the CRC control plan. Long-term cancer control in LMIC could be adapted using the step-ladder pyramidal approach.

## Introduction and background

Colorectal cancers (CRC) are the third leading cause of cancer incidence and the second leading cause of cancer mortality globally [1]. In 2020, there were an estimated 1.9 million new CRC cases, with 935,000 deaths attributable to CRC [1]. Recent studies show that 65% of colorectal deaths occur in Asia [1,2]. A higher CRC incidence is observed in countries undergoing economic transition. This rise in CRC incidence is attributed to changing dietary patterns and secondary lifestyles leading to increased animal-source foods and obesity [1]. A significant proportion of CRC mortality is avoidable by CRC screening [1,3]. There is high-quality evidence, mainly from high-income countries, that population-based screening programs for CRC effectively reduce incidence and mortality [4].

Many countries, especially low- and middle-income countries (LMICs), are trying to implement CRC screening programs to improve CRC outcomes [5,6]. The European Guidelines for Quality Assurance in Colorectal Cancer Screening and Diagnosis recommend an organized population-based screening [7]. Subsequently, cancer control program implementation is emerging in North and Central Asian countries (NCAC) [8,9]. Many NCAC have CRC screening programs, including Estonia (since 2016), Georgia (2006), Kazakhstan (2011), Latvia (2009), Lithuania (2009), Russia (non-population-based since 2010), and Ukraine since 2002. Most screening programs utilize noninvasive testing in the form of fecal immunochemical tests (FIT) or fecal occult blood tests (FOBT) annually or biennially, with most programs reporting testing at the regional level [9]. However, many screening programs that screen for breast, colon, and cervical cancer do not achieve their full potential [4]. The efficacy of screening programs is measured by cancer stage, case fatality, survival rates, and disease incidence rate (if the screening leads to the diagnosis of precancerous lesions, e.g., adenoma in CRC). Also, the cancer-specific death rate in the population invited for screening could also measure the success of a screening program. Other important factors include the acceptability of the interventions, quality of life, and cost-effectiveness of the entire program [10,11].

The CRC screening programs face obstacles in the form of low screening compliance due to patient socioeconomic status and knowledge of CRC screening, barriers in provider-patient communication, health insurance coverage issues, and the distribution of endoscopists [12]. Furthermore, forwarding prescreening CRC test information, following up on the completion of testing, conveying results to participants, and coordinating further tests for those who test positive adds up to the logistics of the CRC screening, leading to low and non-sustained compliance [13]. The use of advance notification letters, regional monitoring of results, and further testing could help overcome these challenges. Thus, screening programs facing low participation lead to low efficacy and sustainability [12-14]. Many approaches focusing on individuals, populations, and health systems have been implemented to overcome this obstacle. The 'barriers to effective screening tool' (BEST) and 'soft systems analysis' are some measures proven to improve population-based cancer screening [5]. Studies on cervical and breast cancer screening have shown that population awareness of screening programs improves participation [15,16]. The role of population awareness in improving health is far-reaching, especially for diseases affecting the population at large, sensitive conditions requiring one-on-one discussion, and diseases where barriers exist in seeking care. Discussing CRC-related symptoms or screening tests may have cultural implications, and the possibility of a cancer diagnosis with screening, especially without appropriate prescreening awareness information for participants, could also create fear, contributing to lower CRC screening [17]. Hence, community health awareness programs (CHAP) about CRC and its screening could increase CRC screening uptake [18].

This scoping review aimed to assess community awareness about CRC screening and prevention in NCAC to facilitate cancer control policies. Our findings could assist policymakers in designing effective population-based CRC screening programs in North and Central Asian countries, where the incidence of CRC is rising.

## Review

Methods

Literature Search Strategy

With guidance from a medical librarian, we conducted a systematic search for the literature review until May 2020. We searched PubMed/Medline, Embase, and the Cochrane Library. The search strategy included the following NCAC terms and medical subject headings: Armenia, Azerbaijan, Belarus, Estonia, Georgia, Kazakhstan, Kyrgyzstan, Latvia, Lithuania, Moldova, Russia, Tajikistan, Turkmenistan, Ukraine and Uzbekistan, Colon cancer, Rectal cancer, and Colorectal cancer. Two authors, R.P. and M.A., independently reviewed the identified abstracts for eligibility for the study, and disagreements were settled by consensus. Full details of the search strategy can be obtained from the corresponding author.

Study Inclusion and Exclusion Criteria

We limited our search strategy to studies with English abstracts. We included studies with the following criteria: (1) any of the following study designs: randomized controlled trials (RCTs), cohort studies, case-control studies, observational studies, conference abstracts, and studies with a pre-post design; (2) studies conducted in at least one of the NCAC of interest; (3) studies on CRC screening or prevention-related population awareness and barriers to CRC screening. Specifically, we aimed to summarize: (1) awareness of the availability of CRC screening and modalities for CRC screening, (2) awareness of the symptoms of CRC, (3) knowledge of CRC risk factors, and (4) other barriers to CRC screening amenable to community health education. We excluded studies that were: (1) non-human studies; and (2) case reports.

Article Selection, Data Extraction, and Analysis

**Figure 1:**
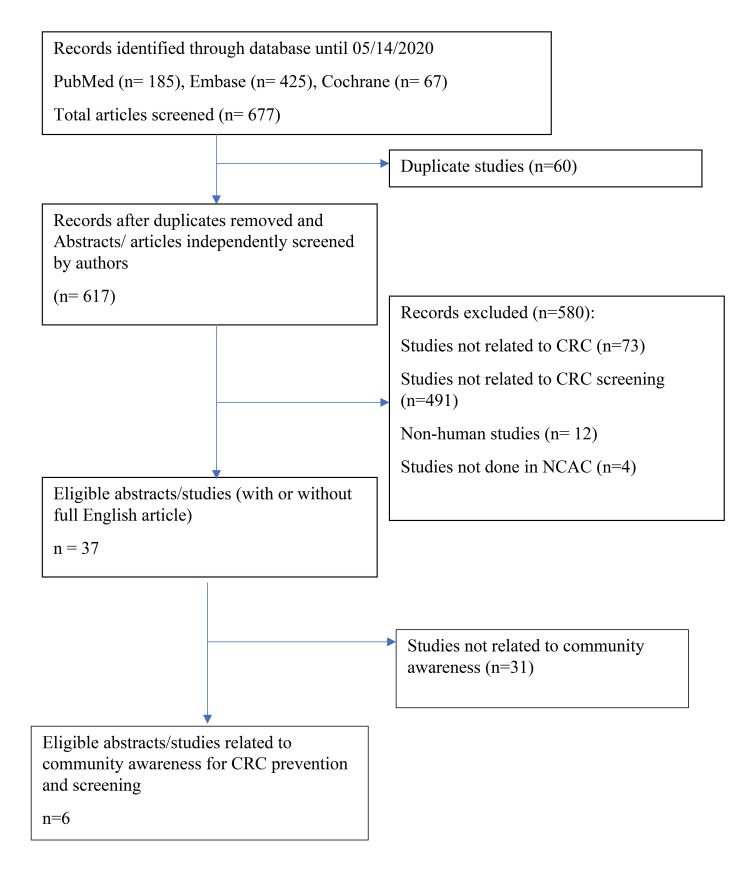
PRISMA flow diagram PRISMA: Preferred Reporting Items for Systematic Reviews and Meta-Analyses

Articles were deemed relevant for inclusion by initially screening the titles and abstracts. If the criteria were not easily identifiable from the title and abstract, the article was included, and a full review was conducted to confirm eligibility. Eligible studies were examined for study location and relation to CRC screening and prevention-related community awareness. After the initial review, further study abstracts, with or without the full article, were included in the review. Figure 1 shows the Preferred Reporting Items for Systematic Reviews and Meta-Analyses (PRISMA) flow diagram.

Data on study characteristics (country, sample size, sex, and gender distribution) and identified facilitators or barriers to CRC screening were extracted and summarized in a table. In addition, identified themes across the included studies were summarized. This review involves non-human subject research and is hence exempt from ethical approval.

Results

We identified 677 publications through the database search until May 14, 2020. After the screening, we included six eligible studies in our analyses. Three of the included studies were full manuscripts [5,13,19], and three were conference abstracts [20-22]. Table 1 summarizes the study characteristics and the major community awareness factors that may positively or negatively affect participation in CRC screening. Further, the strengths of the programs and the barriers are listed.

**Table 1:** Studies related to community awareness of CRC in NCAC CRC: Colorectal cancer, NCAC: North and Central Asian countries, FOBT: Fecal occult blood test

Authors	Type of Study	Sample size	Average age/gender	Country	Community awareness factors with positive CRC outcomes	Barriers associated with negative CRC outcomes	Healthcare system issues	Policy implications
Torosian et al. [19]	Survey population-based cluster sampling	368	55±7.06 Male (46%)	Armenia	Knowledge of CRC; early detection has better outcomes. Would undergo screening if it cost less.	Almost 50% of those concerned about having CRC did not see a physician. Females are more likely afraid to be screened	None reported	Free of cost or subsidized screening tests; improving CRC symptom awareness; awareness focusing on women's health
Turnbull et al. [5]	Health policy analysis	Not reported	Not reported	Estonia	Multiple channels for awareness. Good culturally acceptable information	Unawareness of screening program. Fear of subsequent colonoscopy. Provider barrier for quality assurance. Fatalistic attitude (treatment won't work).	Poor adherence to guidelines and lab quality. Long delays to subsequent investigations and treatment	Need to improve community awareness about the availability of CRC screening programs. Provide appropriate health information about colonoscopy. Maintaining quality metrics for colonoscopy. Increasing access to CRC treatment at a low cost.
Kojalo et al. [20] Abstract	Survey	1191 out of 8149 questionnaires, Participation rate of 14.7%	Not reported	Latvia	Patients more likely to participate in screening: people with screening recommended by primary provider, mee their provider multiple times a year	Patients more likely not to participate in screening: 70-74 age group, rural area, no opinion about screening	Screening program and test information	Involvement of primary care providers to give CRC screening and testing information to patients. Improving CRC screening access in rural areas
Santare et al. [13]	Survey	5129 out of 6023 questionnaires	Participation rate of 85.1%	Latvia	Symptomatic patients more likely to complete screening	Asymptomatic patients are not inclined to screen. Some patients though symptomatic with alarm symptoms did not complete screening	None reported	Improving awareness that CRC could be asymptomatic in the early stages
Yakov T et al. [21] Abstract	Survey	675	Mean age 58.6, men (303)	Uzbekistan	None reported	Inadequate oncological alertness, CRC diagnosis, treatment-related cost, discomfort, unaware of FOBT, distrust in physicians, hesitancy to see oncology	None reported	Increasing awareness about FOBT testing. Involvement of primary care providers to facilitate patient screening and completion of follow-up for abnormal testing.
Poskus et al. [22] Abstract	Survey	289	Not reported	Lithuania	Awareness of screening programs through primary providers, media, and specialist	Not interested in knowing the screening results. Some are unaware of the availability of CRC screening program	None reported	Involvement of primary care providers to facilitate patient screening and completion of follow-up for abnormal testing

The risk of bias was low in two of the three full studies and moderate in one of the studies because of recruitment by convenience sampling from a health facility with low male participation. From the limited data available for the remaining abstract-only studies, the risk of bias seems low to moderate, mostly contributed by selective sampling, healthy user bias, and recall bias. However, a full risk of bias evaluation was not completed for the abstracts. Please see Table 2 for full details.

**Table 2:** Risk of bias assessment

Studies	Sampling bias	Representation bias	Exclusion bias	Non-response bias	Neutral response bias	Social desirability bias
Turnbull et al. [5]	+	-	-	NA	-	-
Santare et al. [13]	Unclear	-	-	-	-	-
Torosian et al. [19]	+	+	-	Unclear	-	+
(+) bias present, (-) Bias not present, Unclear if bias present						

Awareness of the Availability of CRC Screening or CRC Screening Methods

Three studies examined if the target population for CRC screening was aware of the availability of the test or methods of CRC screening. The first study was among residents aged 50 to 74 in two regions in Lithuania that piloted a public CRC screening program [22]. The authors reported that 59% of the participants were aware of the screening program. Among those aware of the CRC screening program, 59% were informed by a physician, while 39% got information through advertisements in the media. Of the eligible respondents who were aware of the availability of CRC screening, only 46% participated in the screening [22]. A second study among patients diagnosed with CRC in Uzbekistan sought to identify obstacles to early CRC detection [21]. None of the 675 patients in the study were aware of FOBT for CRC screening. Of note, Uzbekistan had no organized CRC screening program at the time of the survey.

The third was a cross-sectional study among 384 adults, aged 40 to 64 years, in Armenia [19]. Although 84% of the study population had CRC awareness and 91% reported that early detection improves outcomes, only 22% were aware of CRC screening methods. There was a positive correlation between the level of education or income and the knowledge of CRC screening methods. Only 24% of respondents reported that a healthcare provider had discussed CRC screening with them.

Knowledge of CRC Risk Factors

The study by Torosian et al. [19] evaluated knowledge of CRC risk factors and individuals' perceptions of their susceptibility to CRC. The most commonly endorsed CRC risk factors were genetic factors (76%), low-fiber diet (73%), and smoking (54%). They also found a positive family history correlated with individuals reporting a high susceptibility to CRC. The impact of increased age, obesity/sedentary lifestyle, and alcohol abuse were, however, not evaluated.

Other Barriers to CRC Screening Amenable to Community Health Awareness

A survey conducted in Latvia determined factors associated with non-participation in a population-based CRC screening [20]. In the study, not having a general practitioner (GP) showed the highest odds for non-participation in CRC screening, but with a wide confidence interval (OR 28.6, 95% CI 2.8-295.3). Other identified factors were rural residence (OR 1.7, 95% CI 1.1-21.) and inadequate information about CRC screening (OR 1.7, 95% CI 1.1-2.7). In another study among clinic patients in Armenia [19], only 7% of the survey population reported being advised to get CRC screening by a health provider, with 76% of survey participants reporting willingness to participate in CRC screening if their doctor recommends it. In the same study, 78% indicated a desire to participate in CRC screening if it costs less than $20 or at no charge. Although 39% of the study population believed CRC is a severe illness irrespective of the stage of diagnosis, 23% reported being afraid of CRC screening. Still, the cause of fear was not addressed in the study. Additionally, 44% of the study population believed their risk of developing CRC was low. The most common reasons for not wanting CRC screening were: not thinking screening was necessary (60%), having no symptoms (45%), and cost (41%).

Turnbull et al. evaluated system-based barriers to implementing CRC screening in six European countries [5]. The major system-based barriers identified were the absence of complete population-based registers to identify individuals at risk who may benefit from CRC screening, inadequate resources to implement population-based screening programs, and poor follow-up of individuals with abnormal screening results. The authors also reported that some individuals feared subsequent colonoscopy (if screening tests were abnormal) as a barrier. In Estonia, communities from lower socioeconomic status and ethnic minorities were less likely to participate in screening [5].

The mode of the CRC screening test was shown to influence the uptake of CRC screening in a study in Latvia [13]. The use of the fecal immunochemical test (FIT) resulted in a higher uptake than the guaiac fecal occult blood test (FOBT). In the same survey, sending an advance notification letter before the test kit improved uptake in a subset of patients who received the FOB-Gold test.

None of the studies addressed CRC awareness among government officials, physicians, or cancer screening organizations in these countries. It is difficult to deduce if CRC unawareness amongst this population is also affecting CRC control plans and policies in the region.

Discussion

We found few studies that evaluated community health awareness related to CRC in NCAC. Among identified studies, potential barriers included low awareness about CRC screening, poor perception of CRC risk, and concern about the cost implications. System-based factors such as the availability of CRC screening programs, the availability of comprehensive population registers, and resources to facilitate CRC screening and follow-up of patients who require colonoscopy, were identified as barriers across NCAC [5].

Colorectal cancer has a prolonged precancerous phase, and most lesions diagnosed during screening are precancerous, and their prompt treatment could prevent the progression to CRC [12]. While we found the knowledge of CRC and CRC risk factors to be relatively high in the few available studies, the low knowledge about CRC screening and erroneous perceptions of CRC risk could hamper the success of CRC screening programs. Hence, targeted public awareness programs are needed to enhance the uptake of CRC screening. These awareness programs could focus on providing information about CRC being asymptomatic in the early stages, sporadic in prevalence, and could be seen without a family history of colon cancer. Further information about CRC risk factors and their mitigation to prevent CRC could be provided. Awareness programs focusing on CRC symptoms, alarm symptoms, screening programs, and testing available in the region, with the possibility of availing the test at low or no cost to those with cost barriers, should be implemented. Significant involvement of primary care providers in disseminating the screening program, informing participants about testing quality metrics, and improving physician-patient communication about screening tests would further increase CRC screening uptake.

Other populations have demonstrated low awareness of CRC risk factors, CRC screening modalities, and poor perception of CRC risk [23-25]. In Saudi Arabia, a survey conducted five years after the initiation of the national CRC screening program showed that only 33% of the surveyed population was aware of CRC screening [23]. A Korean qualitative study found a low perceived risk of CRC among participants who were unrealistically overoptimistic about not being vulnerable to CRC because they did not have first-degree relatives with CRC and had healthy lifestyles [17]. In our review, 44% of respondents in a survey of a population at risk for CRC in Armenia considered themselves at low risk for CRC [19]. The CHAP could address these misconceptions about participation in CRC cancer screening [26].

However, there is scarce data about the effect of CRC CHAP on CRC detection and outcomes in LMIC. A Center to Reduce Cancer Health Disparities initiative for promoting CRC awareness in an underserved population in the United States led to 82% of the participants completing and obtaining their CRC screening results in the three-month post-follow-up period [27]. Another study found that an educational intervention aimed at improving CRC screening was effective in improving knowledge of CRC and led to increased uptake of CRC screening [28]. Importantly, the effect of the intervention was found to be durable, with the benefit persisting for up to 12 months after the intervention [28]. Evidence shows that to be effective, CHAP should have clear objectives, a strong evidence base, be culturally sensitive, and aim to influence positive health behavior [27,29,30]. Another challenge to CRC screening is the two-step process, with an initial noninvasive FIT followed by a colonoscopy. The LMIC may not have the resources to ensure patients with a positive screening test proceed to get a colonoscopy. Also, the cost of CRC screening may be a barrier. If screening is available free of cost or at a subsidized cost, it may possibly improve screening uptake. Hence, CRC screening in a true sense, according to international guidelines, is a strenuous goal for LMIC to achieve and sustain amid their other priorities, especially since the effects of cancer screening on disease rates may need decades to be detected.

**Figure 2:**
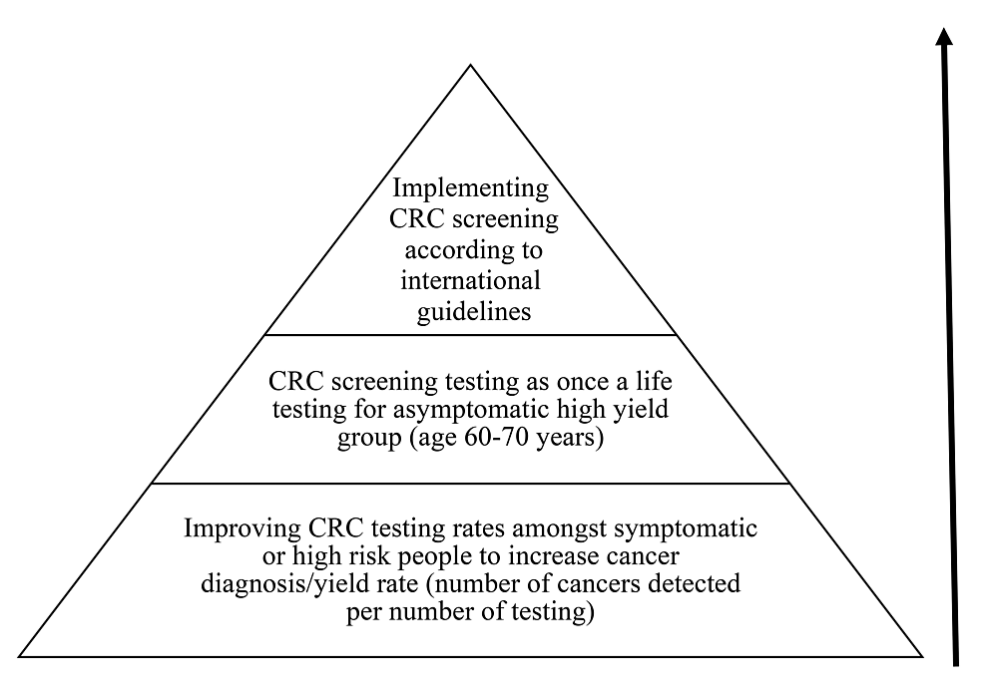
Proposed approach for CRC control implementation programs in LMIC CRC: Colorectal cancer, LMIC: Low- and middle-income countries

Based on our findings and evidence-based programs in the literature, we propose a step-ladder pyramidal approach for CRC control implementation programs in NCAC (Figure 2). This approach could also be extended to other cancer screening programs in other LMIC. As the first step, community awareness and resources should be allocated to increase CRC testing rates among symptomatic or high-risk people, including people with a family or personal history of CRC, to increase the cancer diagnosis yield rate (number of cancers detected per number of tests). The CHAP could play an important role in this phase and should be an integral component of a cancer control program. In the next step, CRC screening testing should be introduced as a once-a-lifetime test for the asymptomatic high-yield group (age 60 to 70 years). In the last step, efforts should be made to implement international guideline-based CRC screening with FOBT yearly, FIT every three years, or colonoscopy every 10 years for people in the 50 to 75 age group. The WHO framework also recommends an early diagnosis program rather than a true screening program for LMIC to increase early diagnosis of cancer, thus preventing its related morbidity and mortality [31].

Our study has limitations in the form of not evaluating the quality of some of the included studies since only abstracts of the study were available. Most studies being surveys may have had a selection and recall bias. We did not include 'Asian' as one of our search terms to avoid reviewing a large number of abstracts for eligibility since our focus was only on a few of the countries in the Asian region. For the same reason, we did not include case reports and may have missed some individual factors suggesting CRC unawareness. We included studies with abstracts and no full articles because of the limited availability of eligible studies. Our study could not be registered with the International Prospective Register of Systematic Reviews (PROSPERO) since the data extraction was completed at the time of registration, and PROSPERO does not allow registration of reviews with completed data extraction.

## Conclusions

In conclusion, unawareness of CRC risk factors, symptoms, screening programs, and CRC screening tests was seen across most of the NCAC. Community unawareness is a major barrier to screening program utilization and sustenance. The NCAC should try to implement a CRC control plan, including a CHAP. Long-term cancer control could be adapted using the step-ladder pyramidal approach.

Possible awareness programs in this region, as per the identified barriers to CRC prevention in our study, should include awareness of CRC symptoms, alarm symptoms, the type of screening program and testing availability in the region, and the possibility of availing the test at low or no cost to those with cost barriers. Efforts should be made to involve primary care providers in disseminating the screening program and improve physician-patient communication about CRC screening to increase CRC screening uptake. Closing knowledge gaps that CRC is mostly asymptomatic in the early stage, sporadic in prevalence, and could be seen without a family history of colon cancer would further improve people’s attitudes toward CRC prevention.
